# Digitalization in Breast Cancer Care: Health App Use and Patient Needs—A Cross-Sectional Survey

**DOI:** 10.3390/healthcare14182964

**Published:** 2026-09-11

**Authors:** Lalesu Geiger, Marion Kiechle, Heike Jansen

**Affiliations:** Department of Obstetrics and Gynecology, University Hospital Rechts der Isar, Technical University of Munich (TUM), 81675 Munich, Germany; marion.kiechle@tum.de (M.K.); heike.jansen@mri.tum.de (H.J.)

**Keywords:** breast cancer, mHealth, health apps, digital health, DiGA, cross-sectional survey, sociodemographic factors, psychosocial wellbeing, information sources

## Abstract

**Highlights:**

**What are the main findings?**

- Health app use among breast cancer patients was associated with younger age, while other sociodemographic associations did not persist after adjustment for age, and lifestyle indicators showed no significant relationship with health app use.
- In this cohort, the use of health apps on prescription was near absent (1/190), and physicians remained the dominant medical information source.

**What are the implications of the main findings?**

- Implementation strategies should prioritize age-sensitive onboarding and usability support to reduce barriers for older breast cancer patients and promote equitable digital engagement.
- Given patients’ reliance on physicians for information and the low health app prescription uptake, clinician-facing, evidence-based guidance and implementation support are key to integrating digital tools into breast cancer care pathways.

**Abstract:**

Background: Despite the rapid proliferation of mobile health applications in oncology, real-world uptake, determinants of use, and patient needs among breast cancer patients remain insufficiently described, particularly in the German healthcare context. This study aimed to assess health app use in breast cancer patients and to identify sociodemographic, lifestyle, and psychosocial correlates. Methods: We conducted a non-interventional, cross-sectional, anonymous paper-based survey among adult breast cancer patients receiving outpatient, day-care, or inpatient treatment, or follow-up at a tertiary university hospital (July 2022–January 2023). Descriptive statistics and group comparisons with false discovery rate correction and multivariable logistic regression were used to examine associations between app use and sociodemographic, lifestyle, and psychosocial variables. Results: A total of 202 patients were included (mean age 56.4 ± 13.8 years). Health app users were significantly younger than non-users (51.7 ± 11.0 vs. 64.7 ± 13.8 years, *p* < 0.001, Cohen’s d = 1.08). Age was the only variable that was independently associated with the use of health apps in the multivariable analysis (adjusted OR 0.43 per 10 years, 95% CI 0.29–0.64). Health app use showed no significant associations with indicators of physical fitness or lifestyle, perceived social support, or emotional burden. Health and fitness apps were the most frequently used app category (77.4%). Physicians were the most frequently consulted source of medical information (77.6%), and only one participant reported having received a digital health application on prescription. Conclusions: In this breast cancer cohort, health app use was common and age-associated. Given patients’ reliance on physicians and the near-absent DiGA prescription rate, age-sensitive implementation strategies and structured clinician guidance are needed to support equitable digital engagement in breast cancer care.

## 1. Introduction

Digital health technologies are increasingly embedded in routine care, with mobile health (mHealth) applications (“health apps”) among the most widely used tools to support prevention, symptom monitoring, self-management, and health information seeking [1,2,3]. In oncology, app-based approaches are particularly relevant because cancer trajectories often require prolonged treatment, follow-up, and supportive care. Breast cancer care comprises extended pathways, during which patients may experience fluctuating symptoms, side effects, and psychosocial distress [4,5]. Accordingly, digital tools that enable timely monitoring and tailored communication may contribute to more patient-centered care and improved outcomes [6,7].

A key development in digital oncology is the systematic collection of patient-reported outcomes (PROs). PROs capture symptoms, functional status, and wellbeing from the patient perspective and complement clinical indicators, especially for documenting treatment burden and quality of life [7,8]. App-based PRO monitoring can facilitate more frequent assessments and may enable earlier recognition of clinically relevant deterioration [9,10]. However, potential benefits depend not only on measurement frequency but also on the integration of PRO data into clinical workflows—particularly whether healthcare professionals receive alerts and initiate timely responses [8,9].

Despite the rapid development of breast cancer-specific digital tools, successful implementation requires an understanding of patients’ current digital behaviors, perceived value, and unmet needs [11]. Even well-designed applications may fail to translate into clinical benefit if patients do not adopt them, discontinue use early, or experience limited relevance. The present work therefore aims to assess the current state of patient digitalization regarding (i) the use of health apps in general and (ii) the use of apps specifically developed for individuals affected by breast cancer. In addition, the study seeks to examine the perceived importance of medical apps and the needs of breast cancer patients related to digital support. Based on these findings, solution approaches concerning the use of apps and digital media will be derived that may contribute to improved medical care for breast cancer patients.

Focusing on breast cancer patients is justified by epidemiological and care-related considerations. Breast cancer predominantly affects women; approximately 99% of breast cancer cases occur in women, and roughly one in eight women is diagnosed with breast cancer during her lifetime [12]. Women also tend to use digital health applications more frequently [13,14], and female physicians appear more receptive to digital health tools than male colleagues [15]. Moreover, breast cancer patients are often medically connected for years due to ongoing therapies and follow-up care, creating repeated opportunities for digital support [4,16].

However, app use is likely patterned by sociodemographic, health-related, lifestyle, and psychosocial factors and sociodemographic characteristics may shape access, literacy, and motivation [17,18,19]. Physical fitness and health status may influence whether patients seek digital support or, conversely, face barriers to use [7,11,18]. Lifestyle factors may be associated with app use because many apps focus on tracking and behavior change [1,20]. Psychological wellbeing and context may further relate to adoption, given that digital tools can provide information, reassurance, and self-management support [6,7,21]. Understanding where patients obtain medical information is essential for designing patient-centered digital interventions aligned with real-world information-seeking practices [22,23].

Accordingly, this study addresses the following research questions:

- Which sociodemographic characteristics are associated with health app use among breast cancer patients, with a particular focus on age, relationship status, household composition, children, living situation and employment-related variables, and are such associations independent of age?
- To what extent are indicators of physical fitness and health status (BMI and physical activity) associated with health app use in this cohort?
- Is health app use associated with lifestyle-related behaviors, including dietary habits as well as alcohol and nicotine consumption?
- Is health app use associated with psychosocial factors, including perceived social support, attention to mental wellbeing and work-related stress, and how does emotional burden related to breast cancer relate to the use of breast cancer-specific apps?
- Which sources of information are most consulted for medical topics among breast cancer patients?

## 2. Materials and Methods

### 2.1. Study Design

To investigate the research questions, we conducted a non-interventional cross-sectional study using a one-time, anonymous survey among patients with breast cancer. A paper-based questionnaire was deliberately chosen over an online survey to reduce the risk of selection bias favoring individuals with higher digital affinity.

Study preparation was carried out with input from statistical experts from the Institute for Artificial Intelligence and Informatics in Medicine at the Technical University of Munich (TUM) and in consultation with legal experts from the institutional data protection office and the third-party funding management unit. Based on sample size considerations, a target sample of N = 200 was defined.

The study was approved by the Ethics Committee of the Technical University of Munich (application number: 2022-394-S-SR) and registered on ClinicalTrials.gov (NCT05605184). The project was initially planned as a bicentric study in cooperation with Charité-Universitätsmedizin Berlin which is reflected in the trial registration entry. The study protocol and questionnaire were prepared accordingly and multicentered conduct was approved by the ethics committee. However, due to delays in the processing of the cooperation agreement, the multicentered implementation could not be realized. As the required sample size could be achieved at a single site, the study was ultimately conducted as a single-center study at the TUM Klinikum rechts der Isar (Munich University Hospital, MRI) Technical University of Munich. Aside from the decision to conduct the study at a single center, no other protocol amendments were made.

### 2.2. Setting

Participants were recruited at the Department of Gynaecology (women’s hospital) at MRI. Eligible patients were approached in outpatient, day-care, and inpatient settings during treatment visits or follow-up appointments. Data collection took place over seven months (July 2022 to January 2023).

### 2.3. Sample and Sampling

The study was registered as a descriptive, non-interventional survey. The goal of the survey was to assess the importance of medical apps and the specific needs of breast cancer patients. In addition, development of approaches to improve medical care for breast cancer patients was anticipated. Therefore, the sample size was determined based on the precision of the principal descriptive estimate.

The planned sample size was set to N = 200.

With a sample size of 200, relative response frequencies can be estimated with a precision of approximately ±7 percentage points; that is, the exact 95% confidence interval extends no more than about 7 percentage points to either side of the estimate (least favorable case, *p* = 0.5). With this sample size and a two-sided significance level of 5%, statistical power approaches 80% for detecting small correlations (e.g., r = 0.20) using a z-test [24]. For the categorical analyses performed, this corresponds to a detectable effect of approximately w = 0.20 in chi-square tests with one degree of freedom (required N = 197) [24], whereas analyses with more degrees of freedom or of the small cancer app subgroup were underpowered and are reported as exploratory.

Ultimately, 202 patients were included.

### 2.4. Participants (Eligibility Criteria)

Inclusion criteria were: (1) diagnosis of breast cancer, (2) age ≥ 18 years, (3) sufficient German language proficiency to participate, and (4) ability to understand and follow instructions provided by study staff. No additional exclusion criteria were specified.

### 2.5. Data Collection Procedure and Participant Flow

**Figure 1:**
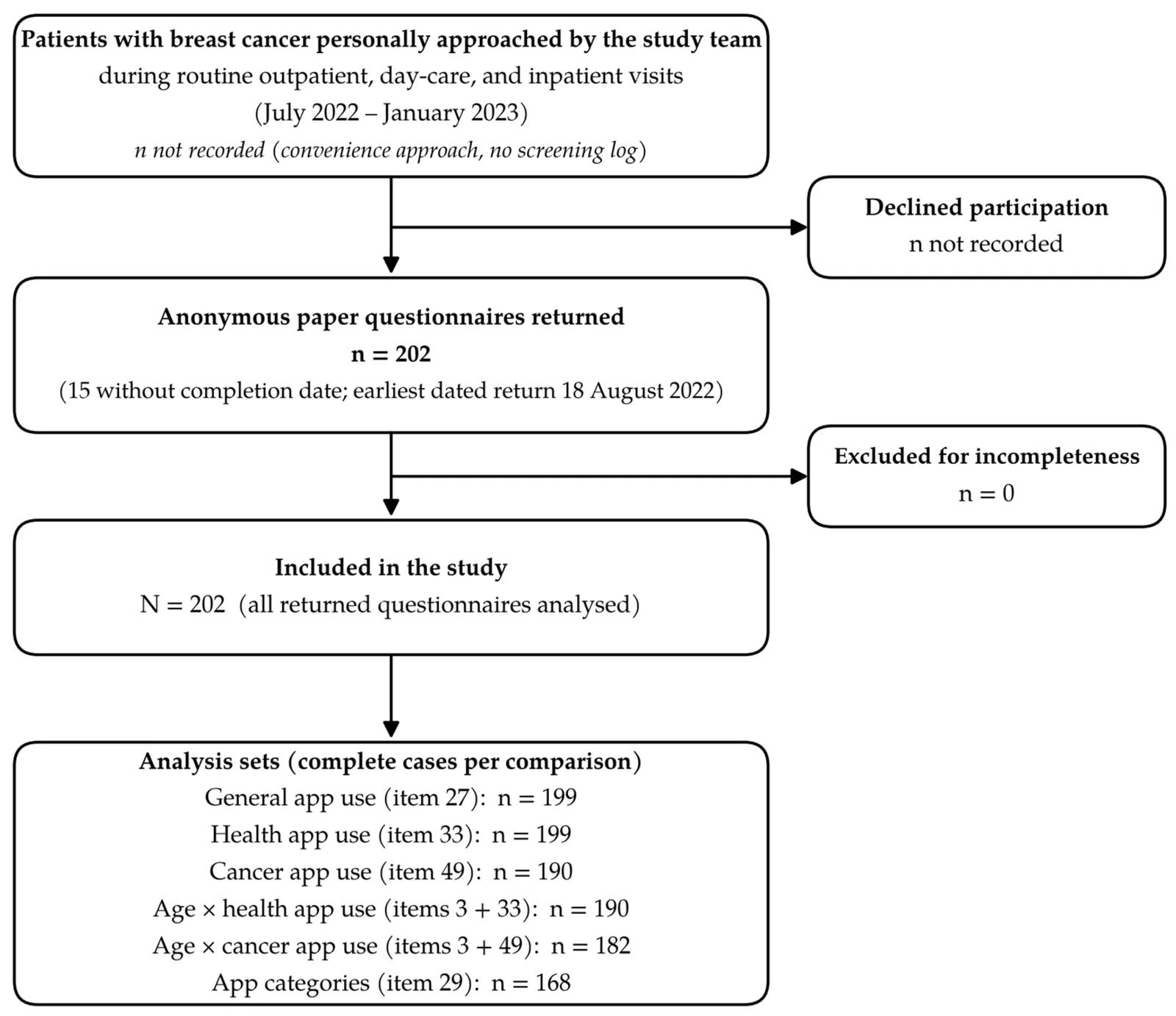
Participant flow and complete-case analysis sets. Item numbers refer to the study questionnaire (Supplementary Material S1). Cancer app use comprises breast cancer–specific and general cancer apps (see Section 2.6).

Data were collected using a paper questionnaire in brochure format. The questionnaire was distributed to eligible patients during treatment visits or follow-up appointments. Participants received information about the study design, the anonymity of the data, and the one-time nature of the survey. It was emphasized that participation was voluntary and that non-participation or individual responses would not affect medical care. The questionnaire was completed independently by participants and required approximately 15 min. Completed questionnaires were returned to staff at the women’s hospital. Recruitment followed a convenience approach: eligible patients were identified and personally approached by members of the study team during routine visits, without a screening log. Patients who declined participation were not registered; consequently, the number of patients assessed for eligibility, the number approached, and the response rate cannot be reported, and selection and non-response bias cannot be quantified. This is acknowledged as a limitation. The reportable participant flow, aligned with the STROBE recommendations, is shown in Figure 1: 202 questionnaires were returned, none was excluded for incompleteness, and all 202 were included. Because item-level non-response varied across items, the complete cases of each comparison are reported in corresponding tables in Section 3.

As the survey was anonymous and completion was voluntary, participants could skip individual items. All 202 returned questionnaires were included; no questionnaire was excluded for incompleteness. The participant flow is shown in Figure 1.

### 2.6. Instruments

The investigators developed the questionnaire de novo specifically for this study. The draft was reviewed and adjusted by the supervising senior clinicians of the study team and a medical statistician and biometrician at the TUM School of Medicine. Formal content validation, cognitive pretests, and pilot studies with patients were not part of the development process, which is acknowledged as a limitation.

The questionnaire contained 53 items of which 52 items covered three domains (1) sociodemographic and clinical characteristics, (2) lifestyle, health status, and psychosocial variables, and (3) media use and health app use. In addition, there was a final item for entering other comments and notes.

The first part (13 items) collected the date of participation and sociodemographic information, including age, sex, place of residence, height and weight, marital status, and living situation. It further assessed whether participants had children, highest educational attainment, employment status, occupation, and perceived economic situation.

The second part (11 items) assessed lifestyle factors, including alcohol and nicotine consumption, physical activity, and nutrition. In addition, psychosocial variables were captured, including perceived social support, work-related stress, and emotional resilience in coping with the disease. Clinical background information included the year of breast cancer diagnosis, therapies received to date, and the presence of comorbidities. To limit respondent burden in a paper-based survey completed during treatment visits, the four psychosocial constructs were assessed by single, purpose-designed items: attention to mental wellbeing (item 21, binary), perceived social support (item 22, binary), work-related stress (item 23, three-level ordinal), and emotional burden related to the disease (item 24, binary; German wording “Fühlen Sie sich durch Ihre Erkrankung emotional aufgewühlt?”, i.e., feeling emotionally upset by the illness). No validated instrument was administered. As reliability coefficients require multi-item scales, no internal-consistency or construct-validity statistics can be reported for these variables. The full instrument is provided as Supplementary Material S1.

The third part (28 items) assessed participants’ media use and health app use. Items addressed internet access, smartphone ownership, general app use and devices used for app-related activities, app categories used, and duration of smartphone use. Participants reported their information sources for medical topics as well as the use, type, and names of health apps. Perceived subjective improvements in physical and/or mental health attributed to health app use were captured. Further items addressed the use of paid apps and willingness to use paid apps. Participants could report whether they had ever been prescribed a digital health application (“app on prescription”, DiGA). Additional questions asked how participants became aware of health apps, which features they considered important, and what they expected from using such apps. Willingness to participate in medical studies via apps was also assessed.

Breast cancer-specific apps were addressed, including use, names of apps used, and perceived subjective improvements in physical and/or mental health associated with breast cancer app use. Open-ended questions allowed participants to describe additional desired functions and content for health apps in general and for breast cancer-specific apps in particular.

Throughout this manuscript, three app use outcomes are distinguished. General app use refers to the use of applications of any kind on a smartphone, tablet, or comparable device (questionnaire item 27: “Do you use apps?”). Health app use refers to the use of one or more health-related applications (item 33). Cancer app use refers to the use of applications addressing (breast) cancer (item 49). Item 49 assessed the use of breast cancer apps. Some participants named apps that were not specifically for breast cancer but were general cancer apps, which were also included in the analysis of this item. Thus, the term “cancer apps” encompasses both breast cancer-specific and general cancer apps. In this manuscript, they are grouped together as “cancer apps.” Each result was analyzed as a binary variable (use vs. non-use).

Self-evident corrections were made according to predefined rules, including the following: If multiple mutually exclusive response options were selected, the overarching or more frequently occurring category was coded (e.g., for weight, smoking, exercise). Logical inconsistencies between filter questions and follow-up questions were resolved in favor of the filter question. Open-ended responses were assigned to existing categories where possible, or in cases of multiple mentions new groups were created for coding where it seemed appropriate. Participants who named a particular cancer app elsewhere in the questionnaire were coded as cancer app users in item 49 (“Do you use one or more apps related to breast cancer?”). Prior reported app use combined with choosing “I do not use health/breast cancer apps” in items 35, 36, 50 and 51 (where respondents were asked whether using such apps improved their health) in the assessment questions was counted as missing, as no concrete statement regarding the subjective assessment is evident in these cases.

### 2.7. Data Analysis

Completed anonymous questionnaires were transferred for coding and analysis into an Excel database (Microsoft Corp., Redmond, WA, USA). During data preparation, self-evident corrections were applied where appropriate (Section 2.6). Participants were categorized into users and non-users of apps, health apps and cancer apps for comparative analyses.

Statistical analyses were performed using IBM SPSS Statistics (version 29.0.0.0, IBM Corp., Armonk, NY, USA). Key analyses, including all regression models and the multiplicity adjustment, were independently re-computed for verification in R (version 4.4.2, R Foundation for Statistical Computing, Vienna, Austria).

Descriptive analyses included frequencies, percentages, means, and standard deviations. Group comparisons were conducted using *t*-tests and chi-square-based tests as follows: Associations between categorical variables were examined using Pearson’s chi-square test. If the expected number of cells in a cell was less than five, Fisher’s exact test was used instead; for tables with more than 2 × 2 cells, the Fisher–Freeman–Halton test was used. When Levene’s test showed equal variances, Student’s *t*-test was applied, and when it pointed to unequal variances, Welch’s *t*-test was used.

Effect sizes are reported throughout: Cohen’s d with 95% confidence intervals for continuous comparisons, odds ratios (OR) with 95% confidence intervals for 2 × 2 tables, and Cramér’s V for larger tables or where an OR was not estimable due to an empty cell.

A two-sided *p* value < 0.05 was considered statistically significant. Since the available data are based on a descriptive study, all group comparisons are exploratory. To control the false discovery rate (FDR), FDR correction was applied, and the central research questions were additionally tested using multivariable analysis.

The Benjamini–Hochberg procedure was applied within each family of tests to limit the false discovery rate resulting from multiple exploratory tests. The families are defined by table. Both unadjusted *p*-values and *q*-values adjusted using the Benjamini–Hochberg method are reported. The *q*-values were calculated from the unrounded *p*-values. Results that were nominally significant but lost their significance after adjustment are marked as such.

Multivariable logistic regression examined whether bivariate associations persisted after adjustment for age. The primary model for health app use included age (per 10 years, forced in), employment status, household composition (collapsed post hoc from seven to four categories owing to sparse cells), and relationship status. Calibration (Hosmer–Lemeshow), Nagelkerke’s R^2^, and collinearity (variance inflation factors) were assessed. As the Box–Tidwell test indicated nonlinearity of the age effect, a sensitivity analysis with categorical age (<50, 50–64, ≥65 years) was performed. For cancer app use, a deliberately minimal two-predictor model (age, emotional burden) was specified, as the number of events supported no more than two predictors at approximately ten events per variable; the same rationale applied to a supplementary three-predictor model for general app use. Regression models were not included in the multiplicity adjustment.

Some participants skipped individual questionnaire items. As analyses were conducted using complete-case data for the respective variables, the number of participants included in each analysis varies and missing data are reported within the corresponding analyses.

Body mass index (BMI) was calculated from self-reported height and weight and classified into the six WHO categories (underweight, <18.5 kg/m^2^; normal weight, 18.5–24.9 kg/m^2^; pre-obesity, 25.0–29.9 kg/m^2^; obesity classes I–III, ≥30.0 kg/m^2^) [25].

The presented analysis in this manuscript focuses on the associations specified in the study objectives.

## 3. Results

### 3.1. Health App Use and Sociodemographic Characteristics

**Health App Use**

A valid response of health app use (item 33) was given by 199 of the 202 participants, of which 126 were health app users (63.3%) and 73 non-users of health apps (36.7%; missing n = 3; cf. Table 1). General app use was reported by 169 of the 199 participants (84.9%; missing n = 3), while the use of (breast) cancer apps was reported by 22 of the 190 (11.6%; missing n = 12).

**Age**

Age was reported by 193/202 participants (missing n = 9). The mean age was 56.4 years (SD 13.8; range 27–87).

As detailed in Table 1, app users and health app users were significantly younger than non-users. Among participants who used apps in general, health app users also remained younger than those not using health apps (51.7 ± 11.0 vs. 59.5 ± 13.2, *p* < 0.001, d = 0.67).

Due to the small number of patients using cancer apps (n = 22), findings regarding the use of cancer apps are exploratory. The results showed that, while users of cancer apps were generally younger than non-users, this association was not statistically significant when the analyses were limited to general app users (see Table 1).

**Sex**

Most participants were female (193/202; 95.5%); one participant was male (0.5%), and sex was missing for eight participants (4.0%). Due to the very small number of male participants, no further sex-stratified analyses were performed.

**Social environment**

Sociodemographic and social environment variables with their associations with app use outcomes are summarized in Table 2.

Most participants reported being in a relationship (n = 129, 66.5% of valid responses, missing data n = 8) and had children (n = 141, 72.7% of valid responses, missing data n = 8). Relationship status or having children was not associated with app, health app or cancer app use.

Living situation was reported by 192/202 participants: 76.6% lived with others and 23.4% lived alone. Participants living alone were markedly older than those living with others (63.8 ± 13.7 vs. 54.2 ± 13.1 years; *p* < 0.001; Cohen’s d = 0.73, 95% CI 0.39–1.07). Living situation (alone vs. with others) and also household composition were associated with general app use (*p* = 0.001, q = 0.003; *p* = 0.007, q = 0.027; see Table 2), whereas employment status was associated with health app use and general app use (both q < 0.001). To evaluate age-related confounding, age-adjusted regression analyses were conducted, which are presented in Section 3.4. None of these associations persisted after adjustment for age.

In contrast, living alone versus with others was not associated with health app use: When household composition was analyzed in more detail, a nominally significant association with health app use was observed (*p* = 0.025, Cramér’s V = 0.26) as patients living with a partner and children used health apps more often compared to other living constellations (which were categorized as follows: alone; with a partner; with parent(s); with child(ren); with a partner and children; with a partner and parent(s); with a subtenant). However, this result was not significant after FDR correction (*q* = 0.062) and was also not detectable in the multivariable model (see Section 3.4).

### 3.2. Physical Fitness, Lifestyle, Psychological Wellbeing, and Information Sources

**Physical fitness and lifestyle**

Neither app use nor health app or cancer app use was associated with queried lifestyle and physical fitness items (diet, physical activity, BMI, alcohol consumption, smoking; shown in Table 3).

Most participants reported paying attention to a healthy diet (111/200, 55.5% consistently; 83/200, 41.5% sometimes; missing data n = 2). Most participants reported ≥30 min physical activity 1–2×/week (62/191, 32.5%) or 3–4×/week (57/191, 29.8%). BMI was available for 193 participants; most were normal weight (46.6%) or pre-obese (35.8%). Alcohol consumption data were available for 193 participants, of which 51.2% reported no alcohol intake. Smoking status was available for 194 participants, of which 89.2% were non-smokers.

**Psychological wellbeing**

Most participants reported paying attention to their mental wellbeing (179/194, 92.3%) and an adequate social support (182/199, 91.5%), but there was no association shown between these items with app/health app or cancer app use.

Emotional burden due to breast cancer was common (127/194, 65.5%). Although there was shown a nominal association with cancer app use at first (*p* = 0.041; OR 3.50, 95% CI 0.99–12.36), this association was no longer statistically significant after FDR adjustment (*q* = 0.244) and in adjusted analysis (see Section 3.4, Model 2). It must be emphasized that the subgroup of cancer app users was very small, and therefore any conclusions and calculations are limited.

Work-related stress responses were available for 168 participants.

Likewise, work-related stress initially showed an association with general app use (*p* = 0.015), which was corrected after FDR analysis (*q* = 0.178). Work-related stress was not associated with health app use or cancer app use.

The results are shown in Table 4.

**Information sources**

Information sources were assessed regarding medical information (item 32, n = 196) and how patients learn about health apps (item 47, n = 127) using multiple response questions which included an open-ended category labeled “Other”. Responses provided in that category were coded into further categories if they appeared repeatedly. The following percentages refer to the number of participants who answered the respective question, not to the number of responses.

Physicians were the most frequently reported source of medical information (152/196, 77.6%), followed by television (76/196, 38.8%), social media (70/196, 35.7%), books (69/196, 35.2%), and personal experience reports (60/196, 30.6%). All remaining sources were consulted by ≤29.1% (professional journals 57/196, 29.1%; online blogs 45/196, 23.0%; lifestyle magazines 42/196, 21.4%; lectures 28/196, 14.3%), while open text responses mentioned under “Other” included self-help groups or patient organizations, guidelines or scientific publications, radio or podcast programs, and health insurance funds, as well as two responses with no further specification (each category named by ≤4 of 196 participants, ≤2.0%).

When asked specifically how participants learned about health apps, the three most common sources were family and friends (47/127, 37.0%), social media (45/127, 35.4%), and healthcare professionals (42/127, 33.1%). Additional sources included the internet in general (28/127, 22.0%), professional journals (16/127, 12.6%) and television reports (15/127, 11.8%).

All remaining sources were reported less frequently, including flyers or posters, lifestyle magazines, health insurance funds, personal blogs, health apps preinstalled on devices, and three responses without further specification (each named by ≤8 of 127 participants, ≤6.3%).

### 3.3. App Categories, Prescribed Apps, and Self-Reported Impact of Health Apps

Across participants who answered the item on used app categories (168/202, 83.2%, multiple responses allowed), health and fitness apps were the most frequently reported category (130/168, 77.4%, including participants who did not select item 33 but indicated that they use a health app, or who mentioned a specific health app elsewhere in the questionnaire; otherwise only based on answers to item 33: 126/199, 63.3%). After that, social media (124/168, 73.8%) and news apps (110/168, 65.5%) followed. Further commonly used categories included food/drinks/recipes (95/168, 56.5%) and music (89/168, 53.0%). Lower use rates were reported for finance/business (66/168, 39.3%), lifestyle/shopping (65/168, 38.7%), games (60/168, 35.7%), education (53/168, 31.5%), and relaxation/meditation apps (30/168, 17.9%).

Only one participant reported having used a prescribed digital health application (“app on prescription”; 1/190, 0.5%), whereas 189 participants (189/190, 99.5%) reported no such use (missing: n = 12).

Among health app users, most respondents did not perceive a positive impact of health apps on their health status. Of the 112 users who provided information on physical health effects, 70.5% (79/112) reported no improvement and 29.5% (33/112) reported an improvement. Regarding mental health effects, 114 users provided information; 78.1% (89/114) reported no improvement whereas 21.9% (25/114) reported an improvement.

The impact of health app use on health status is summarized in Table 5.

Only 22 patients reported using cancer apps including breast cancer apps.

### 3.4. Multivariable Analyses

In order to assess confounding, multivariable logistic regression models have been fitted. Results are shown in Table 6.

In these models, age remained the only variable independently associated with the use of apps and health apps (Model 0, general app use, aOR 0.26, 95% CI 0.14–0.51, *p* < 0.001; Model 1, use of health apps: adjusted OR 0.43 per 10 years, 95% CI 0.29–0.64, *p* < 0.001).

The bivariate associations between employment status, household composition, and living situation did not remain significant once age was taken into account and relationship status was likewise not independently associated.

Since the Box–Tidwell test indicated nonlinearity in the effect of age (*p* = 0.006), age was additionally modeled categorically (Model 1b). The results remained essentially unchanged, with the steepest decline observed among participants aged 65 years and older (aOR 0.08 compared with those under 50, 95% CI 0.02–0.29).

In addition, an exploratory model for the use of cancer apps was developed (Model 2, 21 users), in which neither age (aOR 0.73, 95% CI 0.50–1.07) nor emotional burden (aOR 2.99, 95% CI 0.83–10.75) reached statistical significance.

## 4. Discussion

### 4.1. Sociodemographic Characteristics and Social Environment in Relation to App Use

In Germany, the mean age at diagnosis for female breast cancer patients has been reported at around 64 years [12]. In our sample, the mean age was 56.4 years (SD 13.8). This comparatively younger age distribution could plausibly be explained by the recruitment setting at the Department of Gynaecology of the TUM University Hospital (Klinikum rechts der Isar), which is embedded in specialized structures for breast cancer care and includes dedicated services for individuals with hereditary risk constellations [26], as hereditary breast cancer syndromes are characterized by earlier-onset disease patterns, which can further shift the age distribution in a tertiary, specialized center [27].

Across the cohort, health app users were significantly younger than non-users. This finding aligns with broader population-level observations that digital health app use is more prevalent in younger age groups [28,29]. Studies that have already been published indicate that an age of ≥65 years is an appropriate cutoff age for defining older patients in the context of eHealth [30,31], consistent with our multivariable finding of the steepest decline beyond 65 years. In sum, our data support age as a key correlate of mHealth uptake, also within a disease-specific context such as breast cancer.

Most participants lived with others, predominantly with a partner, with children and partner, or alone. In our analyses, the dichotomous comparison (living alone vs. with others) was not significantly associated with health app or cancer app use. However, more granular household composition showed a nominally significant association with health app use in bivariate analyses, but did not hold up after correction for multiple testing or adjustment for age. These patterns do not reflect an independent effect of social context but are consistent with the age structure underlying the household configurations as people living alone were significantly older than those living with others. More extensive studies with sufficient statistical power are needed to determine whether certain household structures have an additional, age-independent influence on digital use (see Section 5).

### 4.2. Health Awareness and Lifestyle as Correlates of App Use

In this study, lifestyle- and health awareness-related parameters assessed for statistical associations included BMI, physical activity, healthy dietary behavior, and nicotine and alcohol consumption. None of these variables showed a statistically significant association with the use of health apps or cancer apps. Thus, the assumption that a healthier lifestyle and higher health awareness are linked to the use of health apps and cancer apps was not supported by our data.

One possible explanation is that health apps may be attractive regardless of an individual’s baseline health awareness and lifestyle. This interpretation is consistent with the observation that health apps represented the most frequently reported app category in the cohort (77.4%). Health apps may therefore be used both to maintain health-related routines and to pursue new health goals, rather than reflecting an already established “healthy lifestyle” profile. Under these conditions, health apps may appeal to a broad range of breast cancer patients across different lifestyle patterns. This suggests the potential of app-based approaches as scalable entry points to promote health awareness, balanced nutrition, increased physical activity, and weight normalization.

Adopting a healthy lifestyle has been associated with improvements in quality of life and potentially prognosis among breast cancer patients [32,33,34]. To determine whether health app use can actively facilitate such changes, further research is warranted. Prospective longitudinal studies with larger samples and repeated assessments over time would be suitable to evaluate the directionality and magnitude of potential effects and to estimate the real-world benefit of health apps in this patient population.

### 4.3. Psychosocial Factors and Social Environment in Relation to App Use

We examined whether patients’ psychological wellbeing and social environment were associated with the use of health apps and cancer apps. The variables assessed for statistical associations included social and sociodemographic context factors (relationship status, living situation, and perceived social support) as well as indicators related to psychological health. Overall, no associations were observed between these parameters and the use of health apps or cancer apps. An association between emotional burden due to breast cancer and the use of cancer apps did not withstand FDR correction and age correction, but this signal will be discussed in Section 4.7. Emotional burden was not associated with the use of health apps or general app use in our cohort.

The absence of significant associations suggests that these apps may function as supplementary tools in patients’ everyday lives rather than substitutes for interpersonal care. This interpretation is supported by participants’ open-ended comments, which emphasized that apps cannot replace physician visits or qualified medical personnel and that personal conversations and human support remain essential. Several respondents also raised concerns about misinformation in apps and expressed a preference for hybrid models in which digital tools complement, but do not replace, direct patient–provider contact.

In addition, our analyses did not provide evidence that having children was associated with higher use of health apps or cancer apps.

The lack of statistically significant findings regarding psychosocial and social–environmental variables may reflect methodological constraints, including limited statistical power due to sample size and the cross-sectional design. Nevertheless, these results provide a useful basis for future research. Longitudinal studies with larger samples and repeated assessments over time would be particularly suitable to further clarify whether psychosocial factors influence the adoption and sustained use of digital health applications in breast cancer care.

### 4.4. Health and Fitness Apps as the Predominant App Category

In our breast cancer cohort, health and fitness apps were the most frequently reported app category (130/168, 77.4%), narrowly ahead of social media apps (124/168, 73.8%). This proportion is markedly higher than what has been reported for the general population of smartphone users in Germany, where only about one quarter report using fitness apps in consumer surveys [35,36]. The difference is unlikely to be explained by age alone, as interest in health-related apps is also increasing among older adults, including those aged 65 years and older [37].

A plausible interpretation is that a breast cancer diagnosis increases patients’ engagement with health-related behaviors and self-management, thereby elevating the relevance and perceived usefulness of health and fitness apps compared with the general population. Accordingly, the high prevalence of health and fitness app use in our sample may reflect disease-related informational and behavioral needs, with patients potentially turning to digital tools to support activity, nutrition, symptom monitoring, and broader health maintenance during and after treatment [6,7,11].

### 4.5. Perceived Impact of Health Apps and the Role of Human Support

In our cohort, concerns about losing personal contact with healthcare professionals emerged as a recurring theme. Participants emphasized that digital tools should complement direct communication with physicians and qualified clinical staff. This aligns with the broader notion that mHealth is most acceptable when embedded in hybrid care models, where digital monitoring or information is paired with accessible human support and clear clinical accountability [38,39,40].

Building on this, the self-reported impact of health app use on health status was modest. Only about one in three health app users perceived an improvement in physical health, and about one in five reported an improvement in mental health. These proportions appear lower than those reported in some population surveys, where users often describe health apps as supportive in daily life and as providing reassurance [41]. One potential explanation is that patients with breast cancer face complex, high-burden treatment trajectories [5,42]. General-purpose health apps may therefore have limited capacity to produce noticeable changes in symptoms or wellbeing compared with the needs arising from cancer treatment and survivorship. Another explanation is that the perceived benefit may depend strongly on whether apps are tailored, personalized, and integrated into professional care, which are features that are not consistently present in the broader commercial app market [11,43].

Notably, perceptions of limited benefit do not necessarily indicate that apps are ineffective. Rather, they may reflect mismatches between app functionality and patients’ priorities, insufficient guidance on choosing appropriate tools, or challenges in sustained, meaningful use during periods of high physical and emotional strain. In addition, some patients may experience digital burden, such as feeling constantly reminded of illness or engaging in excessive self-monitoring, which are factors that can reduce perceived usefulness, particularly in psychologically demanding phases of care, as has also been observed in other chronic disease contexts [41,44].

If formally assessed, prescription-only digital health applications were to receive more favorable user ratings than “everyday” health apps, this would suggest that quality assurance, evidence orientation, and clinical relevance may be critical drivers of perceived value. This interpretation supports the need for (i) better guidance for patients and clinicians in selecting appropriate tools, (ii) clearer expectations about what health apps can realistically achieve in cancer care, and (iii) evaluation studies with larger samples and longitudinal designs to determine which app features, use patterns, and care integrations lead to measurable benefit in breast cancer populations.

### 4.6. Limited Uptake of Digital Health Applications on Prescription in Routine Care and Central Role of Clinicians in Breast Cancer Care

The notably low prescription rate of digital health applications on prescription in our cohort indicates that such tools are not yet embedded in routine clinical practice, reflecting trends reported in statutory health insurance monitoring, where uptake varies substantially across regions and over time [13,45,46].

Clinician-related hesitancy appears to be a central factor. While many healthcare professionals express general support for the concept of prescribing DiGA, only a smaller proportion report an intention to prescribe, citing insufficient information, uncertainty regarding evidence, and legal or technical concerns as key barriers [15,47]. These obstacles are mirrored in patient-reported experiences: although more than one in five respondents in a Doctolib survey expressed interest in digital health options, only 2% reported receiving proactive information from their treating physicians [23].

Specialty-specific variation may further shape prescribing behavior. Adoption differs across medical disciplines and may depend on clinicians’ attitudes toward digitalization as well as the availability of suitable, specialty-relevant applications listed in the official DiGA directory [14,48]. This is particularly relevant in breast cancer care: in our cohort, physicians were among the most frequently consulted sources for medical information and also played a key role in how patients first became aware of health apps. These findings are in line with existing evidence showing that patients expect guidance from healthcare professionals when evaluating digital health tools [23,49].

Overall, the results suggest that clinicians act as key enablers in the implementation of evidence-based digital health applications. To support routine use, clinicians require accessible, practice-oriented information, including concise summaries of indications, clinical evidence, data protection standards, and pragmatic prescribing guidance [15,50,51]. While structured resources such as the BfArM DiGA directory already provide detailed information for professional users [48], additional dissemination strategies may be needed to translate this knowledge into routine prescribing behavior.

Professional societies may also play an important role by evaluating mHealth options within their fields and offering quality-assured orientation aids for both clinicians and patients. Future research should therefore investigate which communication formats—such as journal-based updates, targeted newsletters, or dedicated congress sessions—are most effective in improving clinicians’ knowledge, confidence, and prescribing behavior regarding digital health applications on prescription, and should explore specialty-specific barriers to digital adoption in senology or more precisely in breast cancer care.

### 4.7. Use and Impact of Cancer Apps in the Patient Cohort

In our cohort, only 22 participants used cancer or especially breast cancer related apps, limiting generalizability. The analyses shown are therefore hypothesis-generating. In our exploratory analysis, breast cancer app users’ odds of experiencing emotional distress were nearly three times higher, indicating that disease-related psychological strain may be a driver for seeking cancer-specific digital support. As this de novo hypothesis is not statistically significant in our study, further research is needed to evaluate this topic and a potential link between breast cancer app use and emotional distress.

Although almost one third of our health app users reported physical improvement and almost one fifth mental health benefits, external evidence is mixed: some larger studies report more consistent positive effects of digital interventions on either quality of life, self-efficacy, or anxiety [6,7,52,53]. In contrast, the same meta-analysis found no significant effect of using mHealth interventions in breast cancer patients on depression and fatigue [53] and a recent systematic review found no clear evidence that psychological mobile health interventions improve mental health in breast cancer survivors [54]. Discrepancies between our findings and previous studies likely reflect our small number of users and limited digital adoption in Germany [55]. Some participants used apps only for study data entry, which may obscure true health effects. Overall, breast cancer apps are not yet embedded in routine care, potentially due to limited awareness, usability issues, and high patient expectations regarding data security and professional credibility. Improved information from healthcare providers, app developers, insurers, and regulatory bodies may support broader implementation.

### 4.8. Limitations

Several methodological limitations warrant consideration.

The cross-sectional design precludes causal inference, and the single-center recruitment limits generalizability. Self-report bias is an inherent constraint of survey-based research and likely affects variables such as lifestyle behavior and perceived social support. The small subgroup of breast cancer app users and the near-absent DiGA prescription rate further limited statistical power for targeted analyses, and sex-stratified comparisons were not feasible given the near-exclusive representation of women in the sample.

Missing values were concentrated in a small subgroup of participants (eight of the nine participants without age information also did not report their living situation) and missingness was not significantly associated with health app use status (Fisher’s exact test, *p* = 0.176). Nevertheless, residual bias cannot be excluded.

The questionnaire was purpose-built and underwent methodological but not psychometric or patient-based evaluation: no cognitive pretesting or pilot testing was performed. The number of self-evident corrections required during data preparation (Section 2.6) indicates that some items were not consistently understood as intended. The psychosocial variables were measured with single, non-validated items rather than established scales, and no reliability or validity evidence is available for them. Findings involving these constructs should therefore be regarded as preliminary rather than psychometrically established. Two of these items additionally showed marked ceiling effects (perceived social support 91.5% affirmative; attention to mental wellbeing 92.3%), leaving minimal variance. The corresponding null findings may reflect measurement insensitivity rather than a true absence of association.

Future studies should employ validated instruments to assess these constructs reliably.

## 5. Conclusions

This cross-sectional survey examined digital health engagement among breast cancer patients, with a particular focus on the use of health apps and breast cancer-specific applications. Overall, the findings indicate that health app use is common in this cohort, while the use of breast cancer-specific apps appears less frequent, and only one participant reported having received a digital health application on prescription.

Regarding the research questions, age was relevant for app uptake for general app and health apps, whereas the association with cancer app use was purely exploratory given the small subgroup of cancer app users. Health app users were younger than non-users. In contrast, relationship status, having children, and living situation (alone vs. with others) were not robustly associated with health app use. For physical fitness and lifestyle-related factors, no statistically significant associations with health app use were identified for BMI, physical activity, diet, or alcohol and nicotine consumption, suggesting that health apps are used across different health behavior profiles rather than only by individuals with an already “healthy” lifestyle. Regarding psychosocial variables, health app use was not associated with perceived social support, work-related stress or emotional burden due to the disease. As our data is hypothesis-generating in regard to breast cancer patients being more likely to use cancer apps when they are emotionally distressed, there should be investigations conducted in new studies with a reliable sample size. Finally, physicians were the most frequently consulted information source for medical topics, highlighting the central role of healthcare professionals in guiding patients within an increasingly complex digital health landscape.

These findings have practical implications. Age-related differences suggest that implementation strategies should address barriers among older patients. The absence of strong lifestyle gradients further implies that health apps could serve as broadly acceptable entry points. Of note is the central role of physicians as information sources, paired with the near-absent DiGA prescription rate, which underlines the need for structured implementation support for clinicians.

Future studies should build on these findings using multicentered designs with larger and more diverse samples to improve generalizability and statistical power. In the future, larger study groups must be examined to validate findings reported here. Sample estimates of future efforts need to utilize at least approximately 430 subjects at a power of 0.80 and alpha = 0.05, corresponding to ten events per variable for multivariable analysis of the disease-specific outcome, whereas we would recommend a recruitment target of about 500 participants considering possible occurrence of missing data. Longitudinal research with repeated assessments would be particularly valuable to clarify temporal relationships and to evaluate whether health app use can promote sustained improvements in lifestyle behavior, symptom management, adherence, and quality of life in breast cancer patients. In addition, qualitative research could explore patients’ expectations, barriers, and trust-related concerns (e.g., data protection and misinformation) in greater depth. Implementation research should examine how clinicians can be supported most effectively in recommending and prescribing evidence-based digital health tools and how such tools can be integrated into breast cancer care pathways without replacing essential personal contact but rather complementing it in a patient-centered hybrid care model.

## Figures and Tables

**Table 1:** Age comparisons between users and non-users of apps, health apps, and cancer apps.

Comparison	Users (n, Mean ± SD)	Non-Users (n, Mean ± SD)	Age Missing, n (Users/Non-Users)	*p*	Cohen’s d (95% CI)	*q*
App use (overall)	163; 53.6 ± 12.0	27; 72.4 ± 10.8	6/3	<0.001	1.58 (1.14–2.02)	<0.001
Health app use (overall)	123; 51.7 ± 11.0	67; 64.7 ± 13.8	3/6	<0.001	1.08 (0.76–1.39)	<0.001
Health app use (app users only)	123; 51.7 ± 11.0	40; 59.5 ± 13.2	3/3	<0.001	0.67 (0.30–1.03)	<0.001
Cancer app use (overall)	22; 50.0 ± 8.5	160; 56.5 ± 13.9	0/8	0.004	0.49 (0.04–0.94)	0.005
Cancer app use (app users only)	22; 50.0 ± 8.5	137; 53.8 ± 12.3	0/6	0.075	0.32 (−0.13 to 0.77)	0.075

Age in years. Cohen’s d: Based on the pooled standard deviation, reported with 95 percent confidence intervals (CI). *q*: Benjamini–Hochberg-corrected *p*-values in this table (five tests). Age missing: Participants who were excluded from the respective comparison due to missing age data.

**Table 2:** Sociodemographic and social environment variables and their associations with app use outcomes.

Variable	Outcome	n	Effect Size (95% CI)	*p*	*q*
In relationship (vs. not)	Health apps	191	OR 1.83 (0.98–3.42)	0.056	0.093
	Cancer apps	183	OR 3.29 (0.93–11.59)	0.052	0.093
	General apps	191	OR 2.53 (1.11–5.77)	0.024	0.062
Children (yes vs. no)	Health apps	191	OR 0.81 (0.41–1.58)	0.528	0.609
	Cancer apps	183	OR 0.64 (0.25–1.63)	0.343	0.429
	General apps	191	OR 0.90 (0.36–2.27)	0.819	0.819
Living with others (vs. alone)	Health apps	189	OR 1.83 (0.92–3.66)	0.084	0.127
	Cancer apps	181	OR 1.81 (0.50–6.47)	0.575	0.617
	General apps	189	OR 4.26 (1.80–10.09)	0.001	0.003
Household composition (seven categories)	Health apps	189	Cramér’s V = 0.26	0.025	0.062
	Cancer apps	181	Cramér’s V = 0.28	0.049	0.093
	General apps	189	Cramér’s V = 0.33	0.007	0.027
Employed (vs. not employed)	Health apps	191	OR 3.57 (1.92–6.64)	<0.001	<0.001
	Cancer apps	183	OR 2.05 (0.76–5.51)	0.148	0.202
	General apps	191	OR 9.97 (3.29–30.19)	<0.001	<0.001

Analyses were conducted on complete cases; n denotes the number of participants in each analysis. Odds ratios (OR) with 95% confidence intervals (CI) refer to the odds of using the respective app type for the category named first; Cramér’s V is reported for variables with more than two categories or where an OR was not estimable due to an empty cell. Pearson’s chi-square test was used unless any expected cell frequency was below five, in which case Fisher’s exact test or the Fisher–Freeman–Halton test was applied. *q*-values denote Benjamini–Hochberg-adjusted *p*-values within this table (15 tests). Household composition comprised sparse categories (minimum expected cell count 0.1); the corresponding exact tests should be interpreted with caution.

**Table 3:** Lifestyle and physical fitness variables and their associations with app use outcomes.

Variable	Outcome	n	Effect size (95% CI)	*p*	*q*
Diet (balanced nutrition, three categories)	Health apps	198	Cramér’s V = 0.04	0.885	0.916
	Cancer apps	189	Cramér’s V = 0.10	0.320	0.600
	General apps	198	Cramér’s V = 0.09	0.421	0.631
Physical activity (five categories)	Health apps	189	Cramér’s V = 0.18	0.206	0.552
	Cancer apps	180	Cramér’s V = 0.08	0.855	0.916
	General apps	188	Cramér’s V = 0.15	0.369	0.615
BMI (six WHO categories)	Health apps	191	Cramér’s V = 0.13	0.681	0.916
	Cancer apps	183	Cramér’s V = 0.22	0.072	0.409
	General apps	191	Cramér’s V = 0.13	0.916	0.916
Alcohol consumption (three categories)	Health apps	190	Cramér’s V = 0.12	0.257	0.552
	Cancer apps	182	Cramér’s V = 0.13	0.208	0.552
	General apps	190	Cramér’s V = 0.16	0.075	0.409
Smoker (vs. non-smoker)	Health apps	191	OR 0.57 (0.23–1.42)	0.223	0.552
	Cancer apps	183	Cramér’s V = 0.13	0.082	0.409
	General apps	191	OR 1.64 (0.36–7.47)	0.744	0.916

Analyses were conducted on complete cases; n denotes the number of participants in each analysis. Odds ratios (OR) with 95% confidence intervals (CI) refer to the odds of using the respective app type for the category named first; Cramér’s V is reported for variables with more than two categories or where an OR was not estimable due to an empty cell. Pearson’s chi-square test was used unless any expected cell frequency was below five, in which case Fisher’s exact test or the Fisher–Freeman–Halton test was applied. *q*-values denote Benjamini–Hochberg-adjusted *p*-values within this table (15 tests). For smoking and cancer app use, no OR was estimable due to an empty cell; Cramér’s V is shown instead. BMI was categorized in underweight, normal weight, pre-obesity, obesity classes I–III [25]. Sparse categories were present for BMI and diet (minimum expected cell counts 0.5 and 0.7, respectively); the corresponding exact tests should be interpreted with caution.

**Table 4:** Psychosocial variables and their associations with app use outcomes.

Variable	Outcome	n	Effect Size (95% CI)	*p*	*q*
Attention to mental wellbeing (yes vs. no)	Health apps	193	OR 0.87 (0.28–2.65)	0.805	0.879
	Cancer apps	184	OR 0.80 (0.16–3.84)	0.676	0.855
	General apps	192	OR 0.89 (0.19–4.19)	1.000	1.000
Work-related stress (three categories)	Health apps	168	Cramér’s V = 0.16	0.110	0.331
	Cancer apps	164	Cramér’s V = 0.12	0.307	0.527
	General apps	167	Cramér’s V = 0.24	0.015	0.178
Emotional burden (yes vs. no)	Health apps	192	OR 1.58 (0.86–2.93)	0.142	0.340
	Cancer apps	184	OR 3.50 (0.99–12.36)	0.041	0.244
	General apps	193	OR 1.95 (0.88–4.34)	0.096	0.331
Social support (yes vs. no)	Health apps	197	OR 1.21 (0.44–3.33)	0.713	0.855
	Cancer apps	188	OR 0.49 (0.13–1.91)	0.391	0.587
	General apps	197	OR 1.82 (0.55–6.02)	0.300	0.527

Analyses were conducted on complete cases; n denotes the number of participants in each analysis. Odds ratios (OR) with 95% confidence intervals (CI) refer to the odds of using the respective app type for the category named first; Cramér’s V is reported for variables with more than two categories or where an OR was not estimable due to an empty cell. Pearson’s chi-square test was used unless any expected cell frequency was below five, in which case Fisher’s exact test or the Fisher–Freeman–Halton test was applied. *q*-values denote Benjamini–Hochberg-adjusted *p*-values within this table (12 tests).

**Table 5:** Self-reported impact of health app use on physical and mental health status.

Outcome	Respondents (n)	No Improvement, n (%)	Improvement, n (%)
Physical health	112	79 (70.5)	33 (29.5)
Mental health	114	89 (78.1)	25 (21.9)

**Table 6:** Multivariable logistic regression models for general app use, health app use, and cancer app use.

Predictor	Model n (Events)	aOR (95% CI)	*p*
**Model 0: General app use (supplementary model; see footnote)**			
Age, per 10 years	188 (162)	0.26 (0.14–0.51)	<0.001
Employed (vs. not employed)		1.36 (0.31–5.91)	0.686
Living with others (vs. alone)		2.05 (0.71–5.88)	0.182
**Model 1: Health app use (primary model; age continuous)**			
Age, per 10 years	188 (122)	0.43 (0.29–0.64)	<0.001
Employed (vs. not employed)		0.93 (0.39–2.22)	0.870
Household composition (ref. living alone)			0.494
With partner only		0.43 (0.12–1.56)	0.198
With child(ren) in household		0.59 (0.17–2.11)	0.417
Other constellations		2.27 (0.17–29.63)	0.531
In relationship (vs. not)		2.37 (0.80–6.98)	0.118
**Model 1b: Sensitivity analysis (age categorical)**			
Age 50–64 years (ref. <50)	188 (122)	0.37 (0.14–0.96)	0.040
Age ≥ 65 years (ref. <50)		0.08 (0.02–0.29)	<0.001
Employed (vs. not employed)		0.99 (0.41–2.41)	0.978
Household composition (global test)			0.676
In relationship (vs. not)		2.33 (0.82–6.62)	0.113
**Model 2: Cancer app use (exploratory minimal model)**			
Age, per 10 years	176 (21)	0.73 (0.50–1.07)	0.105
Emotional burden (yes vs. no)		2.99 (0.83–10.75)	0.092

aOR, adjusted odds ratio. CI, confidence interval. Events denote app users. Model 0 was restricted to three predictors because only 26 general app non-users had complete covariate data (approximately nine events per parameter), and its Hosmer–Lemeshow test indicated limited calibration (χ^2^(8) = 25.6, *p* = 0.001), attributable to near-zero expected counts of non-users in the upper risk deciles given the very strong age effect. Adjusted estimates from this model should therefore be interpreted as supplementary evidence of age mediation rather than as a calibrated prediction model (omnibus χ^2^(3) = 54.2, *p* < 0.001; Nagelkerke R^2^ = 0.45; no separation). Model 1: omnibus χ^2^(6) = 46.3, *p* < 0.001; Nagelkerke R^2^ = 0.30; Hosmer–Lemeshow *p* = 0.536; all variance inflation factors ≤ 1.69. The Box–Tidwell test indicated a departure from linearity of the logit for age (*p* = 0.006), and results were materially unchanged when age was modeled categorically (Model 1b: omnibus χ^2^(7) = 41.9, *p* < 0.001; Nagelkerke R^2^ = 0.28). Model 2 was specified as a two-predictor model because only 21 cancer app users had complete covariate data (approximately 10 events per variable). It is exploratory. Model 2: omnibus χ^2^(2) = 7.3, *p* = 0.026; Nagelkerke R^2^ = 0.08; Hosmer–Lemeshow *p* = 0.853; Box–Tidwell *p* = 0.061. Analyses were conducted on complete cases.

## Data Availability

The data presented in this study are not publicly available due to privacy and ethical restrictions, as the dataset contains potentially identifying information. Data may be made available upon reasonable request to the corresponding author, subject to ethical approval. The complete list of self-evident corrections applied during data preparation is available upon request from the authors.

## Supplementary Materials

The following supporting information can be downloaded at https://www.mdpi.com/article/10.3390/healthcare14182964/s1, Supplementary Material S1: Original study questionnaire (German).
