# Supplementary material for: Digitalization in Breast Cancer Care: Health App Use and Patient Needs—A Cross-Sectional Survey
Source: Healthcare (Basel). 2026 Sep 11;14(18):2964. doi: 10.3390/healthcare14182964 (PMC13606034; doi:10.3390/healthcare14182964)
Supplement: Supplementary file 1 [file healthcare-14-02964-s001.zip › healthcare-4485835-supplementary.pdf]

Supplementary Material S1. Original study questionnaire (German).
Digitalization in Breast Cancer Care: Health App Use and Patient Needs — A Cross-Sectional Survey (healthcare-4485835).
The questionnaire was administered in German. English translations of individual items are provided in the manuscript where relevant.

1) An welchem Datum füllen Sie diesen Fragebogen aus?
___________.___________.20________

2) Wie alt sind Sie? __________ Jahre

3) Welches Geschlecht haben Sie?
- ☐ weiblich
- ☐ männlich
- ☐ divers
- ☐ Anderes: ______________________

4) In welchem Bundesland leben Sie?
____

5) Wie groß sind Sie? _____________ cm

6) Wie viel wiegen Sie? ____________ kg

7) Wie ist Ihr Familienstand?
- ☐ ledig
- ☐ in einer Partnerschaft
- ☐ verheiratet
- ☐ verwitwet
- ☐ geschieden / getrennt
- ☐ in einer Lebensgemeinschaft
- ☐ Sonstiges: _____________________

8) Mit wem wohnen Sie zusammen?
- ☐ allein
- ☐ Partner
- ☐ Eltern(-teil)
- ☐ Kind(er)
- ☐ Sonstige: ______________________

9) Haben Sie Kinder?
- ☐ Ja
- ☐ Nein

10) Welcher ist Ihr höchster Bildungsabschluss?
- ☐ kein Abschluss
- ☐ Hauptschulabschluss
- ☐ Mittlere Reife (Realschule)
- ☐ Abitur / allgemeine Hochschulreife (Gymnasium)
- ☐ Fachhochschulreife
- ☐ Lehre / Berufsausbildung
- ☐ Universitäts- / Fachhochschulabschluss

11) Wie ist Ihr derzeitiger Erwerbsstatus?
- ☐ angestellt berufstätig
- ☐ selbstständig berufstätig
- ☐ nicht berufstätig und arbeitssuchend
- ☐ nicht berufstätig und **nicht** arbeitssuchend
- ☐ berufsunfähig
- ☐ in Rente / Pension

12) Welchen Beruf üben Sie aus?
____

13) Wie würden Sie aktuell Ihre wirtschaftliche Lage beschreiben?
- ☐ sehr gut
- ☐ gut
- ☐ zufriedenstellend
- ☐ schlecht
- ☐ sehr schlecht

14) Trinken Sie Alkohol?
- ☐ Ja, regelmäßig
- ☐ Ja, nicht regelmäßig
- ☐ Nein

15) Rauchen Sie?
- ☐ Ja
- ☐ Nein
- ☐ Aufgehört im Jahr: ______________

16) Wie oft sind Sie in der Woche körperlich aktiv (mindestens 30 Minuten)?
- ☐ < 1 x / Woche
- ☐ 1 - 2 x / Woche
- ☐ 3 - 4 x / Woche
- ☐ 5 - 6 x / Woche
- ☐ täglich

17) Wann wurde die Diagnose Brustkrebs bei Ihnen gestellt? _________________

18) Welche Therapie haben Sie bis heute erhalten? (Mehrfachauswahl möglich)
- ☐ Operation

Supplementary Material S1. Original study questionnaire (German).
Digitalization in Breast Cancer Care: Health App Use and Patient Needs — A Cross-Sectional Survey (healthcare-4485835).
The questionnaire was administered in German. English translations of individual items are provided in the manuscript where relevant.

- ☐ Chemotherapie
- ☐ Hormontherapie
- ☐ Antikörper-Therapie
- ☐ Strahlentherapie
- ☐ Sonstiges:

  ____

  ____

19) Sind Sie von einer oder mehreren dieser Erkrankungen betroffen? (Mehrfachauswahl möglich)

- ☐ Allergien (z. B. Lebensmittel, Hausstaub, Pollen)
- ☐ Herz-Kreislauf-Erkrankungen (z. B. Bluthochdruck, koronare Herzkrankheit)
- ☐ Psychische Erkrankungen (z. B. Depression, Angststörung)
- ☐ Entzündliche Darmerkrankungen (z. B. Colitis ulcerosa, Morbus Crohn)
- ☐ Neurologische Erkrankungen (z. B. Migräne, Epilepsie)
- ☐ Atemwegserkrankungen (z. B. COPD, Asthma)
- ☐ Diabetes mellitus
- ☐ Fettstoffwechselstörung
- ☐ Endokrinologische Erkrankungen (z. B. Schilddrüsenüber-/-unter-Funktion)
- ☐ Rheumatologische Erkrankungen (z. B. Rheumatoide Arthritis, Polymyalgia rheumatica)
- ☐ Hauterkrankungen (z. B. Neurodermitis, Schuppenflechte)
- ☐ Rücken-/Knochenerkrankungen (z. B. Bandscheibenvorfall, Osteoporose)
- ☐ Sonstige:

  ____

  ____
- ☐ Nein

20) Achten Sie auf eine gesunde, ausgewogene Ernährung?

- ☐ Ja, konsequent
- ☐ Ja, manchmal
- ☐ Nein

21) Achten Sie auf Ihr eigenes psychisches Wohlergehen?

- ☐ Ja
- ☐ Nein

22) Fühlen Sie sich in Ihrem sozialen Umfeld ausreichend unterstützt?

- ☐ Ja
- ☐ Nein

23) Fühlen Sie sich durch Ihr Berufsleben gestresst?

- ☐ Nein
- ☐ Manchmal
- ☐ Oft

24) Fühlen Sie sich durch Ihre Erkrankung emotional aufgewühlt?

- ☐ Ja
- ☐ Nein

25) Haben Sie Zugang zum Internet?

- ☐ Ja
- ☐ Nein

26) Besitzen Sie ein Smartphone?

- ☐ Ja
- ☐ Nein

27) Nutzen Sie Apps?

- ☐ Ja
- ☐ Nein

28) Falls ja, mit **welchem Medium** nutzen Sie Apps? (Mehrfachauswahl möglich)

- ☐ Smartphone / Handy
- ☐ Tablet
- ☐ Laptop / Computer
- ☐ Smartwatch / Fitnessarmband

Supplementary Material S1. Original study questionnaire (German).
Digitalization in Breast Cancer Care: Health App Use and Patient Needs — A Cross-Sectional Survey (healthcare-4485835).
The questionnaire was administered in German. English translations of individual items are provided in the manuscript where relevant.

- ☐ Sonstiges: ______________________ ______________________

29) Falls Sie Apps nutzen, **welche Kategorien von Apps** nutzen Sie? (Mehrfachauswahl möglich)
- ☐ Soziale Medien
- ☐ Spiele
- ☐ Gesundheit / Fitness
- ☐ Lifestyle / Shopping
- ☐ Finanzen / Business
- ☐ Essen / Trinken / Rezepte
- ☐ Bildung
- ☐ Musik
- ☐ Nachrichten
- ☐ Entspannung / Meditation
- ☐ Sonstige: ______________________ ______________________

30) Wie viel Stunden am Tag nutzen Sie Ihr Smartphone?
- ☐ < 1 h
- ☐ 1 h – < 3 h
- ☐ 3 h – < 5 h
- ☐ 5 h – < 7 h
- ☐ > 7 h
- ☐ Ich nutze mein Smartphone **nicht** täglich.

31) Hat sich Ihr Medien-Konsum durch die Corona-Pandemie gesteigert?
- ☐ Ja
- ☐ Nein

32) Wo informieren Sie sich über medizinische Themen? (Mehrfachauswahl möglich)
- ☐ Zeitschriften (Lifestyle)
- ☐ Fachzeitschriften
- ☐ Soziale Medien
- ☐ beim Arzt
- ☐ Fernsehbeiträge
- ☐ Online-Blogs
- ☐ Bücher
- ☐ Vorträge
- ☐ persönliche Erfahrungsberichte
- ☐ Sonstige: ______________________ ______________________

33) Nutzen Sie eine Gesundheits-App bzw. mehrere Gesundheits-Apps?
- ☐ Ja
- ☐ Nein

34) Falls ja, welche Art von Gesundheits-Apps nutzen Sie? (Mehrfachauswahl möglich)
- ☐ Fitnesstracker
- ☐ Ernährungs-Apps
- ☐ Bewegungs-Apps
- ☐ Schrittzähler
- ☐ Diät-Apps zum Abnehmen
- ☐ Apps zur Terminvereinbarung bei Ärzten
- ☐ Apps für ärztliche Beratung
- ☐ Apps zur Schlafüberwachung
- ☐ Pollenflug-Vorhersage-Apps
- ☐ Krankenkassen-Apps
- ☐ Apps zur Erinnerung an Medikamente
- ☐ Apps zur Überwachung des Impfstatus
- ☐ Apps als Tagebuch zur Erkrankung (Symptome etc.)
- ☐ Medizinische Apps (z. B. zur Auswertung von Blutzuckerwerten)
- ☐ Anti-Stress-Apps
- ☐ Sonstige: ______________________ ______________________ ______________________ ______________________ ______________________

Supplementary Material S1. Original study questionnaire (German).
Digitalization in Breast Cancer Care: Health App Use and Patient Needs — A Cross-Sectional Survey (healthcare-4485835).
The questionnaire was administered in German. English translations of individual items are provided in the manuscript where relevant.

35) Hat sich durch die Nutzung von **Gesundheits-Apps** ihr **körperlicher** Gesundheitszustand verbessert?
- ☐ Ja
- ☐ Nein
- ☐ Ich nutze keine Gesundheits-Apps

36) Hat sich durch die Nutzung von **Gesundheits-Apps** ihr **seelischer** Gesundheitszustand verbessert?
- ☐ Ja
- ☐ Nein
- ☐ Ich nutze keine Gesundheits-Apps

37) Bitte nennen Sie uns die Namen der Gesundheits-Apps, die Sie nutzen:

____
____
____
____
____
____
____
____
____

38) Sind Sie dazu bereit für Gesundheits-Apps zu bezahlen?
- ☐ Ja
- ☐ Nein
- ☐ Nur unter dieser Voraussetzung:

____
____
____

39) Nutzen Sie bereits kostenpflichtige Gesundheits-Apps?
- ☐ Ja, und zwar:

____
____
____

- ☐ Nein

40) Haben Sie von Ihrem Arzt bereits eine App verschrieben bekommen (App auf Rezept)?
- ☐ Ja
- ☐ Nein

41) Falls ja, bitte geben Sie den Namen der verschriebenen App an:

____
____

42) Würden Sie sagen, dass Sie durch die Corona-Pandemie vermehrt Gesundheits-Apps nutzen bzw. genutzt haben?
- ☐ Ja
- ☐ Nein

43) Was ist Ihnen bei der Nutzung von Gesundheits-Apps wichtig? (Mehrfachauswahl möglich)
- ☐ professioneller, medizinischer Hintergrund
- ☐ Layout
- ☐ benutzerfreundliche Bedienung
- ☐ Funktionen
- ☐ geringer Preis
- ☐ Motivation
- ☐ emotionale Unterstützung
- ☐ Fachartikel
- ☐ Datensicherheit
- ☐ Unabhängigkeit von Dritten (wie z. B. Pharmaunternehmen)
- ☐ Möglichkeit zur Personalisierung
- ☐ Sportanleitungen
- ☐ Anleitungen zur Selbstachtsamkeit
- ☐ Kontaktmöglichkeit zu anderen Usern
- ☐ Kontaktmöglichkeit zu professionellem, medizinischem Personal
- ☐ Möglichkeit zur Terminvereinbarung beim Arzt
- ☐ Beiträge von Fachpersonal
- ☐ Erklärung von Fachbegriffen

- ☐ Tracking von Symptomen
- ☐ Erfahrungsberichte
- ☐ Erinnerungsfunktion für Termine
- ☐ Erinnerungsfunktion für Medikamenteneinnahme
- ☐ Sonstiges:

  ____
  ____
  ____
  ____

44) Was erwarten Sie von der Nutzung von Gesundheits-Apps?

- ☐ eigene (Weiter-)Bildung
- ☐ Tracking von Gesundheitsdaten
- ☐ Prävention (Vermeidung) von Krankheiten
- ☐ Verbesserung vorhandener Krankheiten
- ☐ Steigerung des körperlichen Wohlbefindens
- ☐ Steigerung des seelischen Wohlbefindens
- ☐ Motivationssteigerung
- ☐ Austausch mit anderen Usern
- ☐ Kontakt mit professionellem bzw. medizinischem Personal
- ☐ Vermeidung Arzttermine zu vergessen
- ☐ Vermeidung Medikamente zu vergessen
- ☐ Weiteres:

  ____
  ____
  ____
  ____
  ____

45) Würden Sie Gesundheits-Apps nutzen, um an medizinischen Studien teilzunehmen?

- ☐ Ja
- ☐ Nein

46) Würden Sie eher an einer medizinischen Studie teilnehmen, wenn die Daten über eine App erhoben werden?

- ☐ Ja
- ☐ Nein

47) Wie werden oder wurden Sie auf Gesundheits-Apps aufmerksam?

- ☐ Empfehlung von Familie/Bekannten
- ☐ Empfehlung von medizinischem Personal (Ärzten, Krankenpflegern etc.)
- ☐ Soziale Medien
- ☐ Persönliche Blogs
- ☐ Fernsehbeiträge
- ☐ Fachzeitschriften
- ☐ Lifestyle-Magazine
- ☐ Internet-Recherche
- ☐ Flyer / Poster
- ☐ Sonstiges:

  ____
  ____

48) Welche weiteren Funktionen und / oder Inhalte würden Sie sich **für Gesundheits-Apps** wünschen?

____
____
____
____
____
____
____
____
____
____

49) Nutzen Sie eine App oder mehrere **Apps zum Thema Brustkrebs**?

- ☐ Ja, und zwar (Name(n) der App):

  ____

Supplementary Material S1. Original study questionnaire (German).
Digitalization in Breast Cancer Care: Health App Use and Patient Needs — A Cross-Sectional Survey (healthcare-4485835).
The questionnaire was administered in German. English translations of individual items are provided in the manuscript where relevant.

____
____

- ☐ Nein

50) Hat sich durch die Nutzung von **Brustkrebs-Apps** ihr **körperlicher** Gesundheitszustand verbessert?

- ☐ Ja
- ☐ Nein
- ☐ Ich nutze keine Brustkrebs-Apps.

51) Hat sich durch die Nutzung von **Brustkrebs-Apps** ihr **seelischer** Gesundheitszustand verbessert?

- ☐ Ja
- ☐ Nein
- ☐ Ich nutze keine Brustkrebs-Apps.

52) Welche weiteren Funktionen und / oder Inhalte würden Sie sich **für Apps speziell zum Thema Brustkrebs** wünschen?

____
____
____
____
____
____
____
____
____

53) Sonstige Anmerkungen / Notizen etc.

____
____
____
____
____
____
____
____
____

____
____

Vielen Dank für Ihre Teilnahme an dieser Umfrage!
